# A LCMS Metabolomic Workflow to Investigate Metabolic Patterns in Human Intestinal Cells Exposed to Hydrolyzed Crab Waste Materials

**DOI:** 10.3389/fbioe.2021.629083

**Published:** 2021-02-15

**Authors:** Fionn Ó Fearghail, Patrice Behan, Niklas Engström, Nathalie Scheers

**Affiliations:** ^1^School of Chemical and Pharmaceutical Sciences, Technological University Dublin, Dublin, Ireland; ^2^Division of Food and Nutrition Science, Department of Biology and Biological Engineering, Chalmers University of Technology, Gothenburg, Sweden

**Keywords:** LCMS, metabolomic, workflow, Caco-2, cells

## Abstract

We have developed a LCMS metabolomic workflow to investigate metabolic patterns from human intestinal cells treated with simulated gastrointestinal-digested hydrolyzed crab waste materials. This workflow facilitates smart and reproducible comparisons of cell cultures exposed to different treatments. In this case the variable was the hydrolysis methods, also accounting for the GI digestion giving an output of direct correlation between cellular metabolic patterns caused by the treatments. In addition, we used the output from this workflow to select treatments for further evaluation of the Caco-2 cell response in terms of tentative anti-inflammatory activity in the hopes to find value in the crab waste materials to be used for food products. As hypothesized, the treatment identified to change the cellular metabolomic pattern most readily, was also found to cause the greatest effect in the cells, although the response was pro-inflammatory rather than anti-inflammatory, it proves that changes in cellular metabolic patterns are useful predictors of bioactivity. We conclude that the developed workflow allows for cost effective, rapid sample preparation as well as accurate and repeatable LCMS analysis and introduces a data pipeline specifically for probe the novel metabolite patterns created as a means to assess the performing treatments.

## Introduction

At present 70% of European shellfish biomass remains as excess material every year. This equates to the same mass as sixty fully laden container ships. This 1.5 mega tonnes of material is all of the parts that are not commonly consumed, mainly consisting of shells, tails and unused meat from the most common European shellfish waste streams, especially that of brown crab (*Cancer pagurus*). At present most of this material ends up going either to landfill, to incinerators or is dumped directly back in to the sea. Although this material is currently treated as a waste product, it is in fact a rich source of a range of useful substances (Bord, 2019; Morris et al., 2019). The leftover meat from shrimp and crab processing is an excellent source of dietary proteins and beneficial fatty acids (Langille et al., 2012; García-Romero et al., 2014). Similarly, the minerals that make up the outer surface of the shells present a source of cheap horticultural stimulants for replenishing mineral contents of soils and for providing nutrients to plants (Sharp, 2013). Therefore, there is a lot of interest lying in the extraction, functionalization and application of each of these different fractions in a range of industrial and consumer sectors including nutraceuticals and food supplementation, fertilizers, coagulants, horticultural stimulants, and non-fossil fuel based plastic (Shigemasa and Minami, 1996; Gildberg and Stenberg, 2001; Kandra et al., 2012; Philibert et al., 2017).

The key to adding value to the commercial shellfish waste streams is to be able to separate the constituent components of interest in an affordable, clean and reliable way. Extraction techniques specifically for the isolation of chitin which apply chemical treatment are well documented as well as techniques that employ the use of commercial enzymatic treatments (Jung et al., 2007; Rhazi et al., 2008; Tarafdar and Biswas, 2013; Hayes and McKeon, 2014; Hamdi et al., 2017; Pighinelli, 2019). However, there is much room to expand on these approaches, especially when seeking to extract value from all of the waste material and therefore utilizing all of the products of the extraction. Application of commercial enzymes and previously unused bacterial fermentation methods seek to produce not just high-quality chitin but also bioactive peptide mixtures (Hayes et al., 2008; Sorokulova et al., 2009; Arbia et al., 2013; Younes et al., 2014; Hajji et al., 2015; Ma et al., 2020). During the isolation of chitin from shellfish waste streams by enzymatic and fermentative processes a range of byproducts are yielded. These byproducts are as yet unexplored sources of bioactive proteins, fatty acids and sugars (Harnedy and FitzGerald, 2012). In seeking to add value to shellfish waste streams these byproducts offer a potential source of dietary supplementation and food additives. As a first step for evaluation of these byproducts, we were using a simulated human gastrointestinal digestion model which simulates mouth, stomach and small intestinal digestion, in conjunction with human intestinal cell epithelia (Caco-2) to investigate potential bioactive effects by hydrolyzed crab waste materials (Glahn et al., 1998; Scheers et al., 2014). The aim was to establish a metabolomic workflow to analyze the metabolic pattern caused by cellular processing of the materials and to differentiate the patterns characteristic of each treatment and to identify which treatment type that produces the most different metabolic pattern from the reference.

As this type of matrices are relatively unexplored, especially in the context of metabolomics analysis, a bespoke sample preparation protocol was required, in order to set a baseline approach for repeatable and accurate analysis of these materials. We found a general knowledge gap in the literature surrounding the handling of products from shellfish waste streams by enzymatic and bacterial treatments. With regards to the LCMS analysis of the cell culture after interaction with the crab hydrolyzates, there was especially a lack of previous work detailing the sample preparation necessary for producing representative, repeatable, and useful data from the analysis. Hence, the task at hand. In this work, we cultured the cells on Transwell^®^ inserts to allow for basal export of metabolites across the basolateral borders, resulting in four distinct fractions of interest for analysis, as metabolites from each digested crab hydrolyzate passed through the cell culture. These fractions were (1) the hydrolyzed raw materials, (2) the apical medium containing the simulated GI-digested hydrolyzates (this is what the cells were exposed to), (3) lysed cells containing whatever was absorbed from the digests, and (4) basal secretions containing metabolic products exported from the cells across the basolateral border (from cellular interactions with the absorbed digests).

We also investigated if the metabolomic patterns could be associated with a pro- or anti-inflammatory cellular response to see if there were any possible biological implications of the patterns caused by the different treatments which would indicate the usefulness of using LCMS metabolomics as an analytical step evaluating potential bioactivity of food or feed materials going through a combined simulated GI digestion/cellular absorption model.

## Materials and Methods

### Samples

The crab waste stream material was sourced from Irish Fish Canners Ltd. Enzymatic treatments of samples named NF1, NF2, NF3, NF4, NF5, and NF6 was carried out at Nofima AS, in Tromsø, Norway by subjecting the crab waste stream material to each of the enzymes in Table 1, under the conditions specified by the manufacturers. The resulting hydrolyzates were lyophilized and stored at −80°C. The sample named HYD was prepared in the University of Ghent, Belgium by treating the crab waste stream material with Alcalase at 1% (v/w) with a substrate/water ratio of 1:4 at 52°C for 2 h. Sample was stored in the liquid phase at −80°C. The crab waste stream material was also fermented by the marine bacteria *Pseudoalteromonas arctica* DSM 18437T (sample 4423) and *Pseudoalteromonas issachenkonii* LMG 19697T (sample 4454) at the University of Ghent. The starter cultures were made by inoculating 100 μl of each strain in 10 ml marine broth at 21°C for 72 h, to reach a final concentration of 10^7^ CFU/ml. Thereafter, 1 ml of starter culture was added to 10 ± 0.02 g of the homogenized crab materials in 40 ml sterile distilled water containing 2% (w/v) NaCl and 0.1% (v/v) acetic acid for 72 h at 21°C, with agitation. The fermentation was terminated by heating the samples at 98°C for 10 min. The collected media were filtered with a piece of gauze and then centrifuged at 2,200×*g* for 20 min. The supernatants were collected and stored at −80°C.

**TABLE 1:** Samples, controls and their designations.

Enzyme used for hydrolysis	Sample designation
Corolase 800 (AB Enzymes GmbH)	NF1
Viscozyme (Novozymes A/S)	NF2
Phosphoserine phosphatase (Royal DSM)	NF3
Carboxypeptidase (CPP) (Royal DSM)	NF4
CPP (pH adjusted to optimum activity) (Royal DSM)	NF5
Viscozyme (pH adjusted to optimum activity) (Novozymes A/S)	NF6
Alcalase (Novozymes A/S)	HYD
**Bacterial strain used for hydrolysis**	
*P. Arctica*	4,423
*P. Issachenkonii*	4,454
**Controls**	
LC mobile phase blank	Blank
Cell Control	C1
Cell digest control	C1Dig

*All samples went through a simulated GI-digestion step before fed to the Caco-2 cells.*

### Cell Culture and Experimental Setup

#### Simulated Gastrointestinal Digestion of Hydrolyzates

The hydrolyzates (2 ml) were added to an alpha-amylase solution (5 ml) containing NaCl (140 mM), KCl (5 mM), alpha-Amylase (105 U/ml) and MQ-water, making 7 ml of simulated oral digest at pH 7. This mixture was incubated for 2 min at 150 rpm agitation at 37°C. To simulate the gastric digestion 1 M HCl was added dropwise until a pH of 2 was reached. Pepsin solution (0.5 ml, 40,000 U/ml in 0.1 M HCl) was added and the volume was brought up to 10 ml [final (pepsin) was 2,000 U/ml] by adding salt solution of NaCl (140 mM) and KCl (5 mM). The gastric digests were incubated at 37°C for 60 min while being agitated at 150 rpm. To simulate onset of the intestinal phase, the pH was raised to 5.5 by adding 1 M NaHCO_3_ dropwise. Then 2.5 ml of bile-pancreatin solution was added, containing 10.6 mg/ml of bile and 7 mg/ml of pancreatin (4 × USP). The pH was again adjusted to 7 by the addition of 1 M NaHCO_3_. Finally, the volume was brought up to 15 ml by adding the salt solution of NaCl (140 mM) and KCl (5 mM). The simulated intestinal digests were incubated at 37°C for 120 min while being agitated at 150 rpm.

#### Caco-2 Cell Culture

Human intestinal Caco-2 cells were purchased from the American Type Culture Collection (ATCC Cat# HTB-37, RRID:CVCL 0025) at passage 17. The cells were continuously cultured (37°C, 5% CO_2_, 95% humidified air) in Minimum Essential Medium (MEM) supplemented with 10% Fetal Bovine Serum (FBS) and Normocin (InvivoGen, San Diego, United States) and seeded between passages 30–35 on Transwell^®^ inserts in 12-well Corning CellBIND plates (Corning, New York, United States) at 100,000 cells/insert. At day 14 post-seeding, the cells were washed in Phosphate Buffered Saline (PBS) and new medium, this time Minimum Essential Medium (MEM) without phenol-red and with 2% FBS, was added to the apical and basal chambers. 24 h later, the basal MEM was aspirated and Hank’s Balanced Salt Solution (HBSS) with 1% FBS was added to decrease the MS signal from the richer medium. Half of the medium in the apical chamber (250 μl) was removed and was replaced by 250 μl of the digested crab hydrolyzate sample (rendering FBS at 1%). The rationale for keeping half of the medium is to maintain cell-produced trypsin inhibitors which protect the cells against the digestive enzymes during the incubation with the digests. The incubation lasted for 4 h at 37°C. All the media containing digests were then aspirated. The basal secretions were taken off and stored at −80°C for analysis by LCMS and the cells were lysed in RIPA buffer (SigmaAldrich) with protease inhibitors (Thermo Fisher Scientific) and were stored at −80°C for LCMS analysis.

#### Statistical Analysis

Three replicates of hydrolyzates were used for the simulated digestion/cell experiments, which were done in triplicate wells at three occasions. Nine samples of cell lysates and basal media was used in the LCMS analyses and three samples were used in the proteomic analysis (data are presented as means ± *SD*). The significance of the differences were analyzed by unpaired, two-tailed, Student’s *t*-test using Microsoft^®^ Excel for Mac, 2020. Differences were considered significant at *p* < 0.05.

### LCMS Metabolomics and Data Workflow

In this study, the consideration for analysis was focused on grouping specific biomarker trends across samples types, treatments types, and controls (Savolainen et al., 2016; Steuer et al., 2019). Software packages such as XCMS (XCMS, RRID:SCR_015538), MetAlign, OpenMS (OpenMS, RRID:SCR_012042) and mzMatch (mzMatch, RRID:SCR_000543) were used to whittle down the LCMS data, setting thresholds and tolerances for intensities and frequencies of specific m/z charge ratios correlated with retention times (Spicer et al., 2017). There are two main approaches employed in metabolomics—targeted and untargeted. Targeted is guided by certain already “known knowns.” Usually, specific molecules of interest with well-defined m/z and retention times are monitored for. This approach is regularly employed when biomarkers are already known, and it is simply their frequency of occurrence and difference in relative abundance between control and treated samples that is of interest. An untargeted approach is much more open ended and poses a whole host of challenges and fundamental chemical and data processing questions that must be considered at every step in the workflow. The analysis is not guided by a known molecule or specific metabolic pathway but rather by the principle of seeking to attain the best snapshot of the biological system in question before, during or after treatment. Thresholds in data analysis are set somewhat arbitrarily, mostly dependent on the quality and limitations of the chemistry of the sample pre-treatment and liquid chromatography (De Vijlder et al., 2018). The major challenge is deciphering what data is useful and what is not. Often in untargeted analysis it can be hard to definitively state the structure of any particular metabolite, even when MS2 scan and precursor/product ion scans are correlated and well matched with databases (Vinaixa et al., 2016). This is due to variation between samples types, between instruments and between pre-treatment protocols. Statistical analysis is applied to whittle down the LC-MS data, so as to highlight the similarities or differences between samples based only on the differing treatments. Peak normalization, ANOVA and Principal Components Analysis are utilized for this purpose. Thusly, the untargeted metabolomics studies are for the most part entirely independent studies, referenced within themselves and interpreted with respect to only the samples, standards and QC analyzed in the same study (Zhao et al., 2018).

**FIGURE 1:**
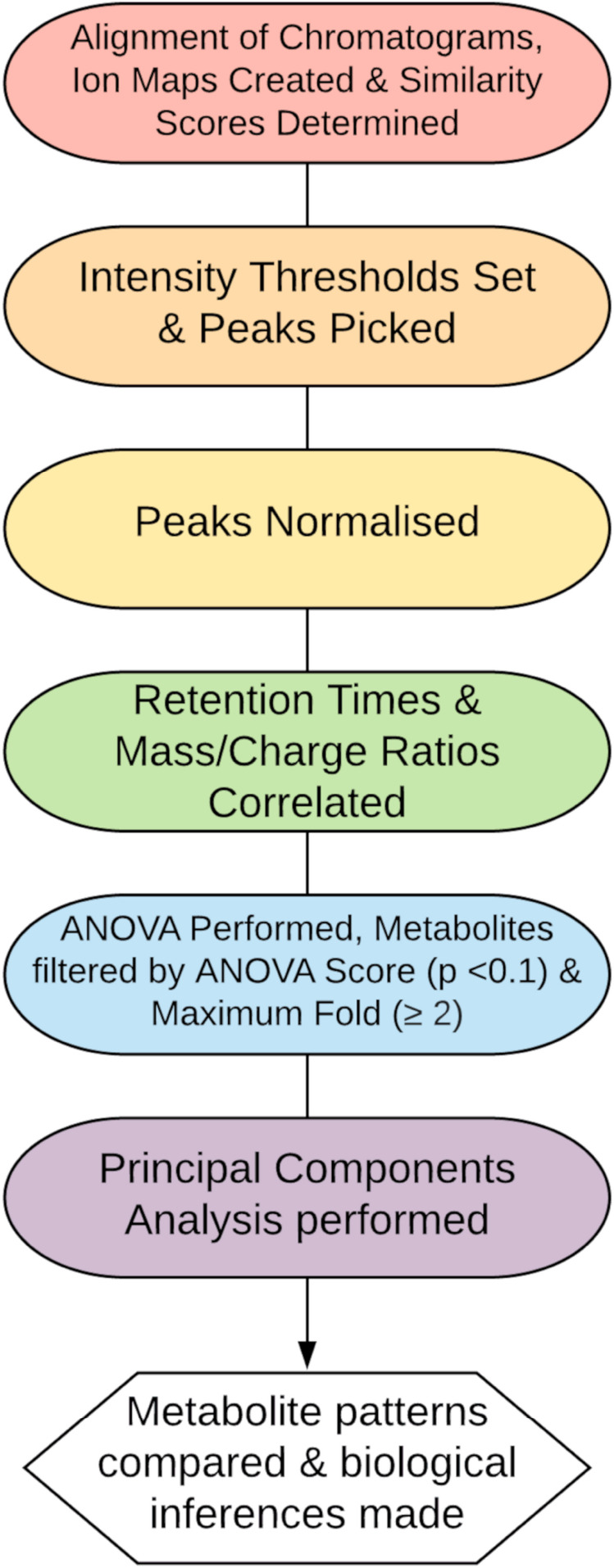
Proposed data workflow for metabolomics analysis.

In this work, we were investigating matrices consisting of a mix with human, crab and sometimes bacterial metabolites and therefore we chose an untargeted approach to examine the metabolite patterns caused by the different crab treatments in comparison to the control cells. From this we could determine the deviation from the controls, which in theory indicate a bioactive effect, and aids in making a decision on which treatment(s) to further evaluate. A bottom up LC-MS workflow design and implementation was developed to analyze the intracellular and basally secreted fraction of Caco-2 cell-produced metabolites and endogenous molecules (Figure 1). Using the Agilent 1260 Infinity II LC system paired with Agilent 6520 Accurate Mass Q-TOF mass spectrometer the fractions of interest were analyzed in both negative and positive ionization mode. A Quadrupole Time Of Flight (Q-TOF) mass spectrometer was utilized in this instance, as in most metabolomics studies, due to high resolution capacity, broad spectral range and high sensitivity.

### Extraction, Pre-concentration, and Sample Preparation

#### Hydrolyzates

Two gram of lyophilized sample was weighed into a 12 ml centrifuge tube. Six milliliter of LC/MS grade methanol was added, and the sample solution was mixed by vortex for 1 min at 800×*g*. The solution was centrifuged at 1,500×*g* for 5 min and the supernatant was transferred into a 25 ml volumetric flask. This procedure was repeated twice and after the third extraction, the volumetric flask was made up to 25 ml with LCMS grade methanol. The sample was filtered using 0.2 μm PES syringe filter and the first 2 ml was disposed of. The sample was then injected into the LCMS. If sample response fell under the signal-to-noise ratio of 3:1 for detection, pre-concentration was performed by TurboVap. Ten milliliter of the filtered extract was placed into a TurboVap flask. The solution was pre-concentrated to 0.5 ml at 40°C with pressure of 2 Bar. 0.5 ml of evaporated sample was placed in a 1 ml volumetric flask. LCMS grade methanol was used to make up to mark. The sample was vortexed for 1 min at 800 × *g* and filtered with a 0.2 μm PES syringe filter before transferring to an injection vial by glass dropper and stored for analysis.

#### Basal Media and Cell Lysates

Samples were centrifuged at 4,000×*g* for 60 min using Amicon Pro 3 kDa Molecular Weight Cut Off (MWCO) centrifuge filters. Filtrates were transferred to a 1 ml volumetric flask and made up to the mark with LCMS methanol. If sample response was above the maximum signal intensity specified in instrument SOP, dilution was applied as needed.

#### Blank and Control Samples

For reference, standardization and to allow for probing of the induced difference in metabolite pattern dependent on each treatment, three control samples were used: (1) The LC blank - prepared using 1: 1 of mobile phase A: mobile phase B, depending on the column and chemistry being used, (2) The cell control: Caco-2 cells not fed with simulated digestion products but which underwent all other sample preparation steps, (3) The digestion control: Caco-2 cells fed with simulated digestion products from a blank digestion with no samples (crab hydrolyzates) in the digestion. The digestion control was used as the reference for comparison of treated samples as it has undergone every step in the digestions, culture and LC procedures except for treatment by enzymatic or bacterial hydrolyzation products. As such, any difference between this metabolite pattern and the metabolite pattern of a treated sample was attributed to the treatment itself.

### LC Parameters, Reagents and Materials

HILIC Mobile Phase Preparation were as follows: Mobile Phase A (10 mM ammonium formate in LCMS water): 50 ml of 100 mM ammonium formate stock solution was made up to 500 ml with LCMS water, transferred to a solvent reservoir and mixed thoroughly. Mobile Phase B (90% ACN with 10% 100 mM ammonium formate): 50 ml of 100 mM ammonium formate stock solution was made up to 500 ml with ACN, transferred to a solvent reservoir and mixed thoroughly. LCMS reagents and materials are listed in Table 2, the instrumentation in Table 3. The Agilent 1260 Infinity II LC system with Agilent 6520 Accurate Mass Q-TOF mass spectrometer was used to perform analysis on all samples, parameters are tabulated in Tables 4–6.

**TABLE 2:** List of reagents and materials.

Reagent/material	Supplier
LCMS methanol	Merck
LCMS acetonitrile	Merck
LCMS Water	Merck
Ammonium formate	Merck
Formic acid	Merck
ACX, Ph(En) and C18 SPE Cartridges	Phenomenex
6 ml SPE tubes	Phenomenex
Amicon^®^ Pro 3 kDa spin tubes	Merck Millipore
0.2 μm PES syringe filters	Merck
Acquity BEH Amide Column, 2.1 × 50 mm, 1.7 μm	Waters

**TABLE 3:** List of instrumentation.

Instrument	Model/make
Liquid chromatography	Agilent 1260 Infinity II LC
Mass spectrometer	Agilent 6520 Accurate Mass Q-TOF
Mass balance	Explorer Ohaus
TurboVap	Caliper Life Sciences TurboVap II
Centrifuge	Beckman GS-6
Vortex mixer	Stuart Vortex SA8
SPE vacuum manifold	Visiprep 24-Port

**TABLE 4:** Optimized HILIC LC acquisition parameters.

LC parameter	Value
Mobile phase	A: 10 mM ammonium formate in LCMS water B: 90% ACN with 10% 100 mM ammonium formate
Column	Acquity BEH Amide Column, 2.1 × 50 mm, 1.7 μm
Flow rate	0.4 ml/min
Injection volume	5 μl
Total run time	11 min
Column temperature	40°C

**TABLE 5:** Optimized LC gradient parameters.

Time (min)	B%
0	5
8	50
8.1	100
11	100

**TABLE 6:** Optimized MS acquisition parameters for both positive and negative ionization modes.

Ms parameter	Value
Gas temperature	300°C
Scan type	Parent Ion
Mass range	0–1,700 m/z
Source	ESI (+ and – polarity)
Dry gas	8L/min
Nebulizer	45 PSI
Collision energy	20 eV
Fragmentation voltage	125 V

### Proteomic Analysis

Assessment of the inflammatory response was carried out by Olink Proteomics (Uppsala, Sweden) Olink employs Proximity Extension Assay (PEA) technology, whereby 92 oligonucleotide labeled antibody probe pairs are allowed to bind to their associated target protein present in the sample. This in turn allows for the formation of a PCR reporter sequence formed by a proximity dependent DNA polymerization event. This polymerization is amplified and detected using real time PCR. The analysis is carried out in 96-well plates without any washing required and allows for in-line replication and quality control samples (Assarsson et al., 2014). 100 μL of basal media samples were submitted for analysis to Olink Proteomics without employing the LCMS sample prep protocol. The PEA technology requires whole proteins in solution for detection and measurements and as such the samples are provided exactly as they are secreted from the cell cultures.

## Results and Discussion

### Optimization of Sample Preparation

There are many tools available for the clean-up of samples and for isolation of specific compounds of interest such as solid phase extraction (SPE), liquid–liquid Extraction (LLE), column filtration, precipitation, and centrifugation. Based on the separation by affinity for a given functional group, stationary phase SPE can allow for large throughput of samples in parallel and cartridges are relatively inexpensive. Analytes of interest are either retained and eluted after interferants have washed free or alternatively analytes are washed through in the first instance while the interferants are retained. Elegant, simple and cost effective, SPE is the analytical standard in many validated protocols (Buszewski and Noga, 2012a; Faraji and Gholami, 2019). Optimization trials were performed with a range of SPE set ups which resulted in either too clean samples or insufficient clean up, giving very noisy and humped chromatograms (data not shown). A different clean up procedure was therefore utilized.

Although undertaken as untargeted analysis, this work had a defined interest in small molecules which are defined as those of a size <1,700 Da (Kalim and Rhee, 2017). As such, we chose to go for size separation instead of affinity-based separation. The intention was not to separate a mixture into its many fractions based on size nor was size characterization of these fractions of interest. Instead simply a cut-off point was needed to isolate the smallest molecules in the samples. Therefore, we used size-based spin filters (Merck Millipore Amicon Pro) with a molecular weight cut off (MWCO) of 3,000 Da. These spin filters allowed for a rapid and cost-effective clean-up of the samples without loss of any small molecules of potential interest.

**FIGURE 2:**
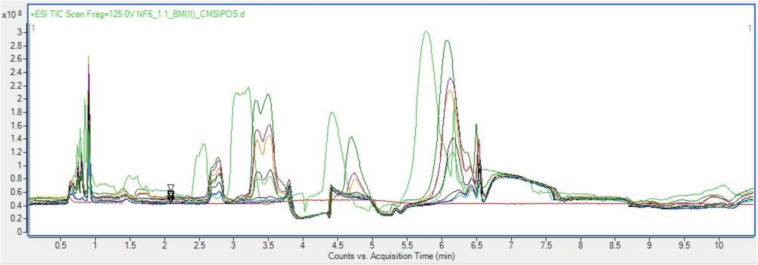
Overlayed positive TICs of Basal media samples run with optimized sample preparation and LC parameters.

Once sample preparation had been optimized to allow for the most representative probing of a given sample, then too was the chromatographic parameters optimized. Based on the occurrence of the bioactive fraction in the aqueous basal medium a focus shifts then to analysis specifically for hydrophilic residues and polar small molecules. As such a Hydrophilic Interaction Chromatography (HILC) column was chosen from the standard metabolomics toolkit for these analyses. HILIC columns combine a range of chemistries to enhance separation of complex aqueous analytes, allowing for a wide spectrum of probing in the space where normal phase chromatography and reverse phase chromatography overlap (Buszewski and Szultka, 2012b; Szykuła et al., 2019). The stationary phase is like that as used in normal phase chromatography, being in this case silica bonded ethylene hybrid (BEH) Amide. Conversely the mobile phase is akin to that used in reverse phase chromatography, in this case acetonitrile and LCMS water. The use of a HILC set up in tandem with the size-based spin filter, sample preparation resulted in clean, well resolved, repeatable chromatograms of each sample as seen in Figure 2.

### Data Analysis and Metabolite Patterns

**FIGURE 3:**
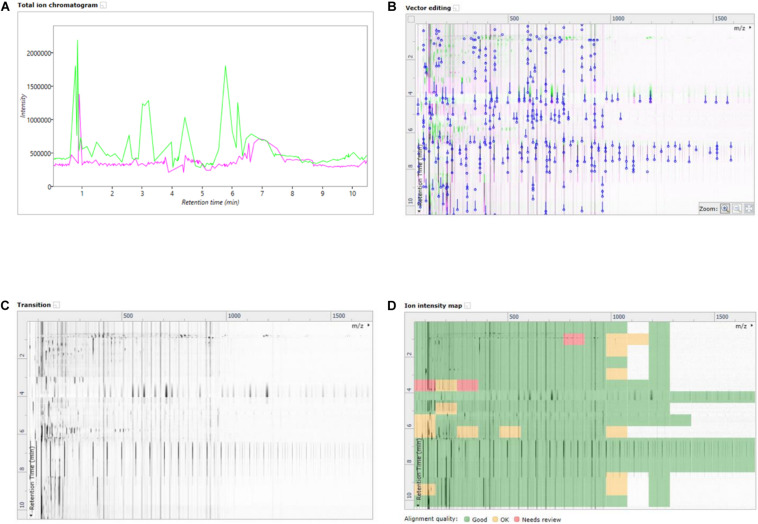
**(A)** Overlayed TICs of NF1 (digested corolase hydrolyzed sample) and C1Dig (digest control). **(B)** 2D overlay of TICs of NF1 (digested corolase hydrolyzed sample) and C1Dig (digest control), with alignment vectors highlighted in blue. **(C)** Alpha blend animation highlighting differences been TICs of NF1 (digested corolase hydrolyzed sample and C1Dig (digest control). **(D)** Ion intensity map of 2D TICs of NF1 (digested corolase hydrolyzed sample) and C1Dig (digest control).

In this work, an emerging visualization scheme was applied which allowed for enhanced interaction with the data without losing the core LCMS elements in the processing. This scheme was the building of ion maps which are two dimensional representations of the chromatograms and mass specs which were layered on top of one another to highlight changes in retentions times, peak intensities and mass/charge signals. The ion map overlay allowed for direct comparison of a treated sample to the reference sample, in a given ionization mode. Seen in Figures 3A–D are the different ion map visualizations from the analysis of the basal media of the Caco-2 epithelia fed with the NF1 (digested Corolase hydrolyzed crab materials). As with all cell lysates or media from the basal compartment, the runs have been aligned with, and are compared to, the reference of C1Dig (Digest control) to account for digestion background effects.

**FIGURE 4:**
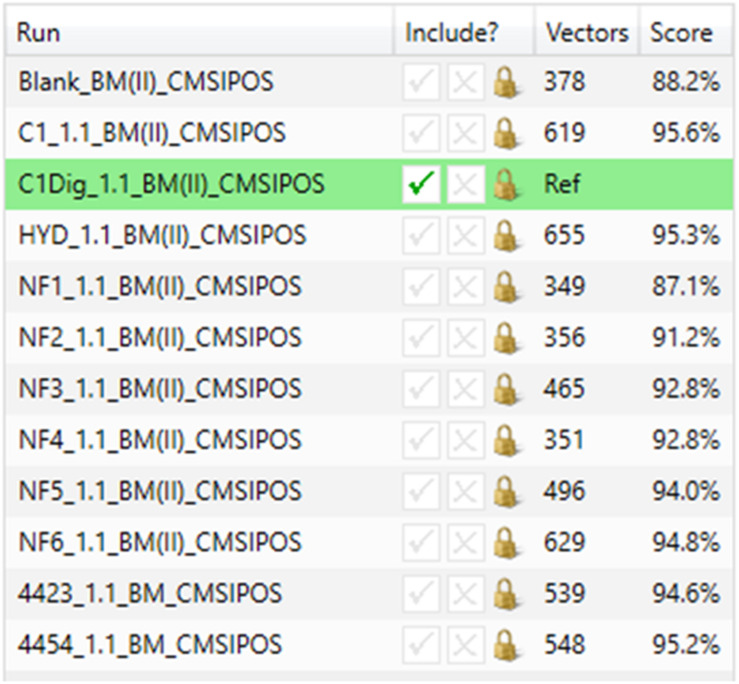
Similarity scores for basal media samples from Caco-2 epithelia in positive ionization mode.

Figure 3A displays the original 1D total ion chromatogram (TIC) as outputted from the LCMS allowing for direct reference to the raw LCMS data, while Figure 3B displays a 2D plot of the sample run in green and the reference run in magenta. The *y*-axis is retention time and the *x*-axis is the mass to charge ratio. The alignment vectors between the common peaks in the sample run and reference are highlighted in blue and are what allow for the alignment of the runs. Figure 3C shows an alpha blend animation, of the same 2D plot, which makes the differences between the sample run and reference run pulse for easy identification. This also helps indicate anywhere that may require manual alignment vectors to be added if missed by the automatic alignment by ProGenisis. Figure 3D contains the ion intensity map of the same 2D plot. This is the most useful portion of the data visualization allowing for a visual quality metric detailing the similarity of the peaks between the sample and reference run. The sections of the map in green are considered significantly similar, yellow sections indicate similarity but with the need for manual review and red indicates poor similarity, based on the calculated similarity scores. The similarity scores which allow for the green, yellow and red visualization within the ion intensity map were calculated based on retention time, peak intensity and extracted mass spectrum. The similarity score is a unitless % score and as such is a direct relative comparison of the retention time, peak intensity and m/z ratios between sample and reference run. Similarity between samples, in this case treatment types, was also established with respect to the reference run. As seen in Figure 4, sample NF3 (PSP) and NF4 (CPP) are as similar to the reference run as one another. Although the treatment applied to the commercial crab waste stream may be different, the extent of biological interaction with the intestinal cells seems to be the same, which is what is specifically of interest in this study.

**FIGURE 5:**
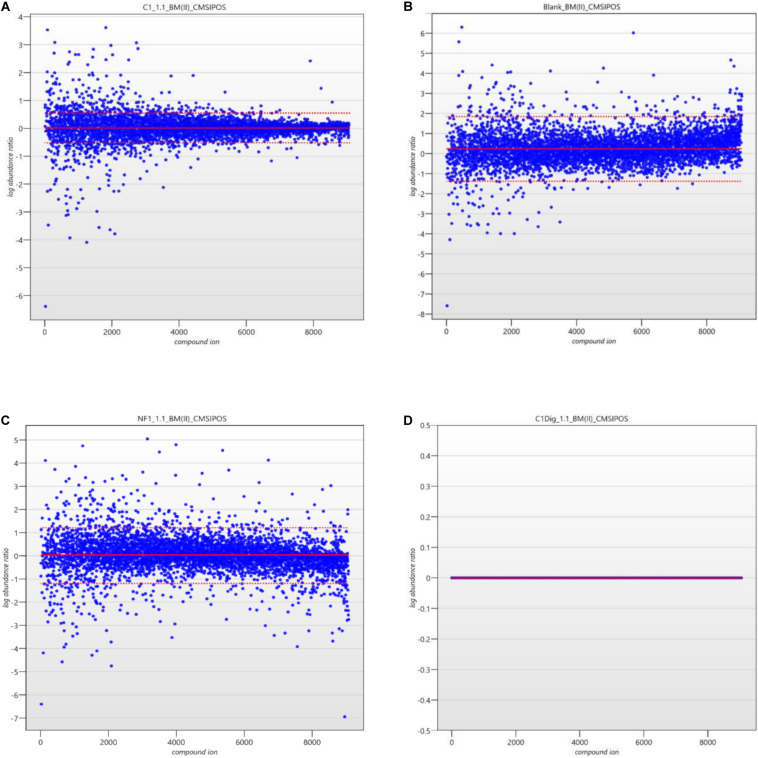
**(A)** Ion normalization plot and limits for C1 (Cell control). **(B)** Ion normalization plot and limits for Blank (LC-MS Blank). **(C)** Ion normalization plot and limits for NF1 (digested corolase hydrolyzed sample). **(D)** Ion normalization plot and limits for C1Dig (Digest Control).

**FIGURE 6:**
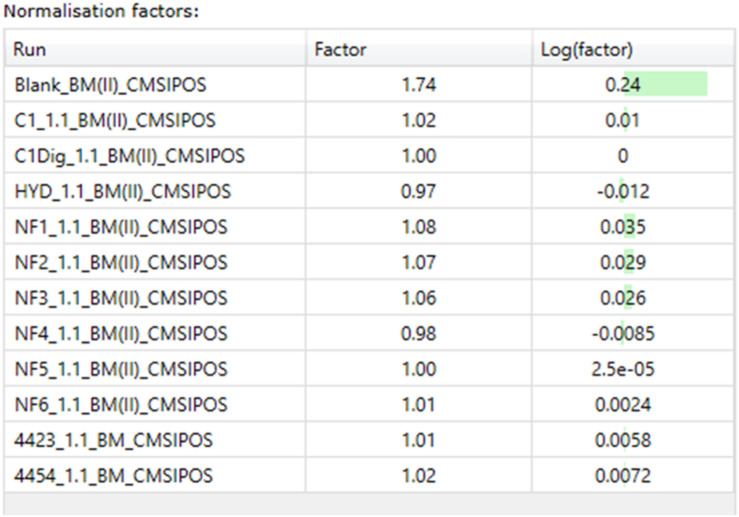
Normalization factors for basal media samples from Caco-2 epithelia in positive ionization mode.

The next step in the data pipeline was peak picking. In untargeted analysis the preeminent approach due to large number of features in a given chromatogram/spectrum is to set peak picking parameters and allow automatic picking. In order to limit the number of features selected, and so as to enhance the ability to identify differences in metabolite patterns between samples, the analytical standard of a 3:1 signal to noise ratio for detection was applied as the threshold for peaks to be picked. Once peaks were picked a normalization was applied in order to remove random and insignificant outliers. As seen in Figures 5A–D, limits were set in relation to the run with the highest number of common peaks across both the samples and the controls. In this case, C1Dig (Digest control) had the most amount of common peaks and as such it’s limits are set to zero and the limits of all other sample are set with respect to this zero point to include as many of the common peaks and to exclude the random outliers. As opposed to referencing specifically the control, this approach ensures that all common peaks, whether generated by the digestion, the treatment or by interaction with the cells, are included and only random outliers are discounted. It also ensures that the degree by which one metabolite pattern differs to another is standardized across samples by applying a normalization factor to each, as seen in Figure 6, allowing for direct comparisons between samples and the controls. This further enhances the ability to isolate features that are different between runs without including random outliers. Without this normalization, outliers would have an unfair weighting in the comparison of metabolite patterns and so runs that are generally the same with a single extreme outlier would be determined to be significantly different from one another, which would not be an accurate representation of their similarities or differences.

Once the peak picking and normalization was complete, each feature was annotated based on retention time correlated with mass/charge ratio. Again, the selection of mass to charge ratios of interest was set somewhat arbitrarily. If all m/z peaks were annotated the data produced would be very noisy with very low specificity. In this case, in order to whittle down the features to allow for good comparison between samples, the three highest abundance m/z ratios at each retention time were selected and correlated. The reason that the three highest abundance m/z signals were chosen is so that each feature correlation is based on a molecular ion peak, a base peak and an alternate high abundance peak at every retention time. This approach is generally applied in targeted quantitative LCMS studies for known compounds and so was applied here as a good starting point for the tentative untargeted metabolomics analysis. It is at this point that statistical analysis is employed in data pipeline, allowing for the comparison between the samples and producing visualizations of the differences and similarities therein. In order to determine significance in the differences between the annotated compounds a Between Subject Design ANOVA was set up whereby samples were grouped by condition into the “Treated Group” and the references as the “Control Group.” This calculation assumed that the conditions are independent and so applies a statistical test of whether the means of each condition are equal. The selected features were then carried forward for comparison with a given database. Progenisis allows for database searching from within the software package itself. Three databases were used; MetLin (METLIN, RRID:SCR_010500), Progenesis Metascope Biomolecules and Human Metabolome Database (HMDB, RRID:SCR_007712) based on best practice from literature (Toldrá and Nollet, 2013; Vinaixa et al., 2016; Spicer et al., 2017; Phelan, 2020).

Once the databases were searched, ~ 1,500 compounds were tentatively “identified” based on the correlated retention time and mass-to-charge ratios. This is a large number of very loosely annotated compounds and so the list was filtered based on two commonly applied criteria. Firstly, only compounds which have an ANOVA *p*-value of <0.10 were considered. It is common to apply a filter of *p* < 0.05 in experiments with much larger data sets, as is common in many large population metabolomics studies. In this case due to the relatively small volume of samples and niche treatment types which have already had the peaks and correlated features screened, a *p*-value of <0.10 was applied to ensure that a good representation of each sample was created. Secondly only compounds with a minimum fold value ≥2 were considered (Kumar et al., 2018). This means that, for a compound to be included in the metabolite pattern, its signal must have occurred with an intensity at least 2 times greater than that observed at the same mass-to-charge ratio in the reference sample. This ensured that a reasonable difference in abundance and frequency must occur for a compound to be carried forward. This ensured that changes in abundance and frequency induced by the instrument, by measurement or by changes in cellular conditions were not taken to be representative of changes induced by treatment. After these filtering criteria were applied, a list of approx. 300 compounds remained. In a study interested in distinct identification and characterization of any of these compounds, they would be carried forward for further analysis by fragmentation at a range of collision voltages, with further MS2 investigations and deeper and more specific comparison with databases.

Eventually a synthetic standard would need to be bought or created for comparison in order to confirm a given compound in the metabolite pattern (Dash et al., 2011; Vinaixa et al., 2016).

However, in this work the interest lied in comparing the metabolite patterns themselves in order to identify which waste treatment procedure produced the most biologically active treatment. As such, the annotated and filtered features were visualized using Principle Components Analysis (PCA) plots. PCA is a common statistical technique which reduces the dimensionality of large complex datasets with multiple variables while maintaining as much variability as possible. This allowed for the comparison of specific sample types with multiple variables in a simple 2D plot. In this case each sample is the hundreds of LCMS features annotated based on retention time and mass-to-charge ratio, filtered by ANOVA scores and minimum fold criteria. The plot shows the spread in differences and the clusters in similarity between samples and controls. This was achieved by solving eigenvector problems, therefore reducing a given feature to a single vector which is taken as a new uncorrelated variable, called a Principle Component. Each feature has successive principle components whereby the variability is successively maximized. As such, by plotting the first PC of each feature against their second PC, a 2D plot of relative variance amongst a data set was produced. In this work, all features were analyzed under the same conditions and all feature data was annotated and filtered the same way using the same units. Thusly, the PCAs produced demonstrate correlation and not co-variance (Tzeng and Berns, 2005; Stein et al., 2006). This means that the difference or similarities between given samples were based on the experimental variable, which in this case was the type of treatment used to produce hydrolyzates of different compositions.

**FIGURE 7:**
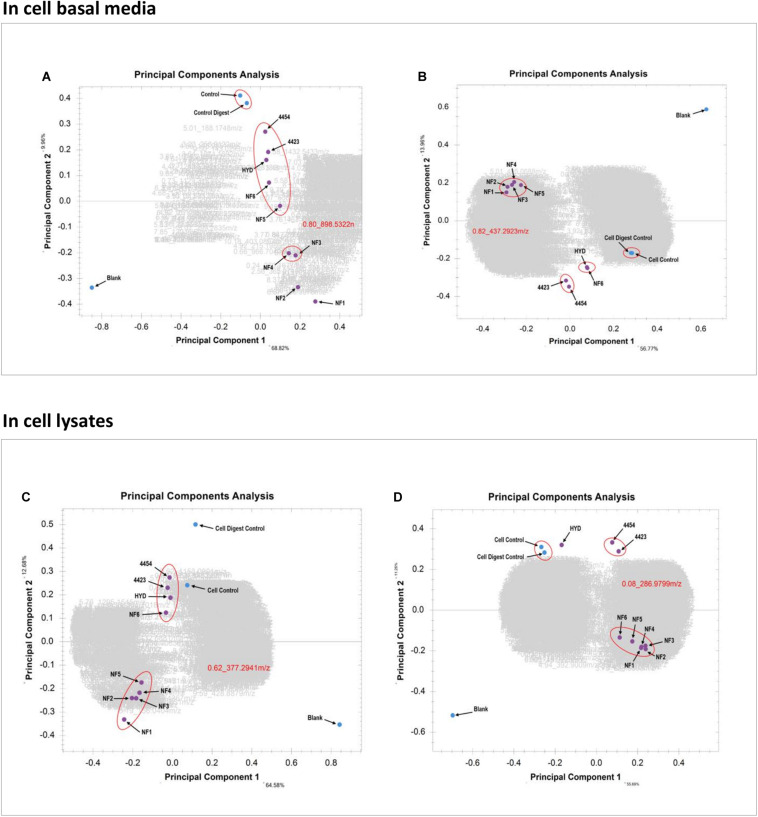
**(A)** PCA of the basal media samples of the Caco-2 epithelia in positive ionization mode. **(B)** PCA of the basal media samples of the Caco-2 epithelia in negative ionization mode. **(C)** PCA of the Caco-2 cell lysates in positive ionization mode. **(D)** PCA of the Caco-2 cell lysates in negative ionization mode.

In Figure 7A the PCA of the basal media samples of the Caco-2 epithelia, in positive ionization mode, is presented. The x-axis contains PC-1, which has the most amount of weighting and the y-axis contains PC-2. It is clearly visualized how different the blank is from all other samples, which was expected. The clusters clearly show the similarity between the two controls as well as the relative similarity between the bacterial treatments and several of the enzymatic treatments. Similarly, samples NF3 (PSP) and NF4 (CPP) are very similar to one another but sufficiently different form the other samples to be clustered alone. In this PCA it is sample NF1 (Corolase hydrolyzed) and NF2 (Viscozyme hydrolyzed) which are furthest from the blank, the controls and the other samples. This indicates that these are the treatments which have induced the greatest biological interaction and thus produced the most different metabolite pattern in the basal media. The PCA of the basal media samples of the Caco-2 cells in negative ionization mode, seen in Figure 7B, further continues the trend of NF1 (Corolase hydrolysis) being the most different sample, as was seen in the positive ionization mode. However, the sample is very tightly aligned and clustered with almost all other enzymatic treatments. Similarly the bacterial fermentation samples were clustered tightly, as are the controls. In the positive ionization PCAs, samples NF6 (Viscozyme, pH adjusted) and HYD (Alcalase) were consistently next to one another but were not only as tightly clustered as the other enzymatic treatments are to one another. Here in the negative ionization basal media PCA NF6 and HYD are distinctly clustered by themselves, demonstrating a very similar metabolite pattern and possibly indicating a similar mode of enzymatic action.

Looking at the PCA of the lysed cells of the Caco-2 epithelia in Figure 7C, the trend of sample NF1 (Corolase hydrolysis) as the most different is continued. However, the difference between NF1 and the next nearest cluster of enzymatic treatments is not as great as in the PCA of the basal media. Similarly, here it seems that the bacterial fermentation treatment samples—4454 (*P. Issachenkonii*) and 4423 (*P. Arctica*)—are clustered together and more closely aligned with the cell control. This PCA indicates strongly that although the internal biochemistry of the cells was affected by the interaction with the different samples, most of the resulting biochemical products and metabolites were secreted from the cells into the basal media. Examining the cell lysate PCA in negative ionization mode, seen in Figure 7D, the trend of most enzymatic treatment samples being clustered persists. However, NF1 (Corolase hydrolysis) is not distinctly the most different sample. Again, the bacterial fermentation samples are clustered as are the controls. Unlike in the negative ionization PCA of the basolateral media samples NF6 (Viscozyme, pH adjusted) and HYD (Alcalase) are distinctly separated. Although the negative ionization basal media PCA indicates a very similar metabolite fraction produced by these treatments, here we see that the sample NF6 has a greater induced effect on the internal cell biochemistry. Perhaps this interaction was producing metabolites that are more persistent and less likely to secrete than those produced by interaction with the HYD sample. The metabolomics data pipeline has outputted distinct, clear, visualized statistical comparisons of the complex metabolite patterns produced from the interaction of each sample at each biological level in the cell culture. From these data, we chose to further evaluate the samples treated with corolase (NF1) and to compare them to the bacterially fermented samples.

### Pro-inflammatory Activity Was Identified

**FIGURE 8:**
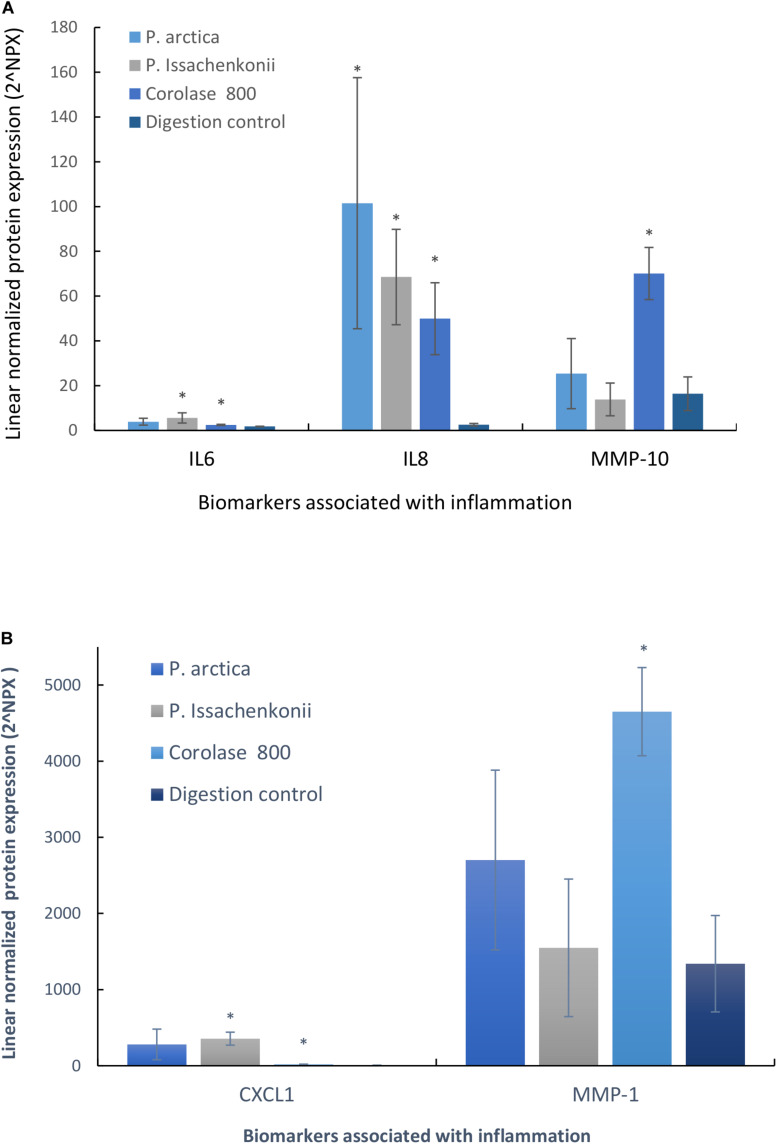
**(A)** Secreted cytokines (IL-6, IL-8, and MMP-10) into the basal media compartment of Caco-2 epithelia in response to hydrolyzed crab materials. **(B)** Secreted cytokines (CXCL-1 and MMP-1) into the basal media compartment of Caco-2 epithelia in response to hydrolyzed crab materials.

As we were interested in finding added value in the crab waste streams, we evaluated the anti-inflammatory potential of the hydrolyzed waste by analyzing the basal media for inflammatory markers (96 biomarkers for inflammation). It was evident that five specific inflammatory protein biomarkers were affected by feeding the Caco-2 epithelia with hydrolyzed crab digests (Figures 8A,B). These were: interleukin-6 (IL-6), interleukin-8 (IL-8), matrix metalloproteinase-10 (MMP-10), matrix metalloproteinase-1 (MMP-1) and chemokine ligand-1 (CXCL-1). IL-6 and IL-8 are specifically pro-inflammatory cytokines which start and maintain inflammatory response to pathogens, wounds and biological stresses. MMP-10 and MMP-1 are enzymes involved in the breakdown of the extracellular matrix which are secreted during reproduction, tissue remodeling and in disease processes, whilst CXCL-1 is a cytokine secreted by melanoma cells associated with angiogenesis, inflammation, wound healing and tumorigenesis. Cellular IL-8 secretion to the basal media was significantly up-regulated in response to all treatments (3,963, 2,675, and 1,949%, respectively, *p* < 0.007, *n* = 3) in comparison to the digestion control, whilst IL-6 levels significantly increased by 315% (*P. Issachenkonii*) and 140% (Corolase 800), *p* < 0.04. Corolase treatment (NF1) of the crab waste, produced significantly increased levels of the metalloproteinases MMP-1 and MMP-10 in comparison to control digests (347%, *p* = 0.003 and 427%, *p* = 0.003, respectively), this effect was not significant for any of the other treatments investigated.

## Conclusion

Utilizing the LCMS metabolomics pipeline, the hydrolyzed crab materials which have the greatest biological interaction with the Caco-2 culture have been identified and compared with the inflammatory response investigations. From the enzymatic and bacterial treatments examined in this work, Corolase 800 indeed can be considered the most effective enzymatic treatment when wishing to produce novel bioactive hydrolyzates from commercial crab waste streams. However, examining the effects of feeding a human GI tract culture model indicates that the hydrolyzates have a negative, pro-inflammatory effect. In seeking to add value to commercial crab waste streams by creating new dietary supplements and nutraceuticals, this work demonstrates that this may not be a likely viable option. However, there are many products used across agriculture, horticulture and botany such as pesticides, antifungals and antihelmintics which seek to create localized inflammation in specific insects and animals. Often these products are synthetic which can mean they are expensive and relatively dirty to produce. Here then possibly lies a way to create natural products which induces oxidative stress in specific insects and animals, without harming crops, soil or water sources. Marine waste materials can be investigated using the same approach by, e.g., using an insect model to evaluate the inflammatory potential after applying this bottom up analytical method for investigating the efficacy of a range of biological extractions. This work allows for cost effective, rapid sample preparation as well as accurate and repeatable LCMS analysis and introduces a data pipeline specifically for probe the novel metabolite patterns created as a means to assess the performing treatments.

## Data Availability Statement

The original contributions presented in the study are included in the article/Supplementary Material, further inquiries can be directed to the corresponding author/s.

## Author Contributions

FF conducted the sample preparation and the LCMS work, metabolomics data analysis, and wrote the first manuscript draft. NE conducted the simulated GI-digestion of the samples and carried out the cell studies. NE and NS analyzed the protein biomarker data (from Olink Proteomics, Sweden). PB and NS supervised the work. PB the metabolomics in particular and NS the digestion/Caco-2 cell studies, in particular. All authors contributed to the article and approved the submitted version.

## Conflict of Interest

The authors declare that the research was conducted in the absence of any commercial or financial relationships that could be construed as a potential conflict of interest.

## Supplementary Material

Supplementary Figure 1 Cell survival (estimated by total protein of live attached cells) and integrity of the Caco-cell epithelia (the tight junction marker; Zonulin) as percentage of digest blank control cells. There was no significant deviation from cell control (digestion blank, 100%) in any case. Data are means ± *SD*, *n* = 3 (Cell survival: *p* = 0.06, *p* = 0.2, *p* = 0.07 and Zonulin: *p* = 0.25, *p* = 0.88, *p* = 0.08, respectively. Changes were considered significant if *p* < 0.05.
