# Supplementary figures and images for: A LCMS Metabolomic Workflow to Investigate Metabolic Patterns in Human Intestinal Cells Exposed to Hydrolyzed Crab Waste Materials
Source: Front Bioeng Biotechnol. 2021 Feb 15;9:629083. doi: 10.3389/fbioe.2021.629083 (PMC7928233; doi:10.3389/fbioe.2021.629083)

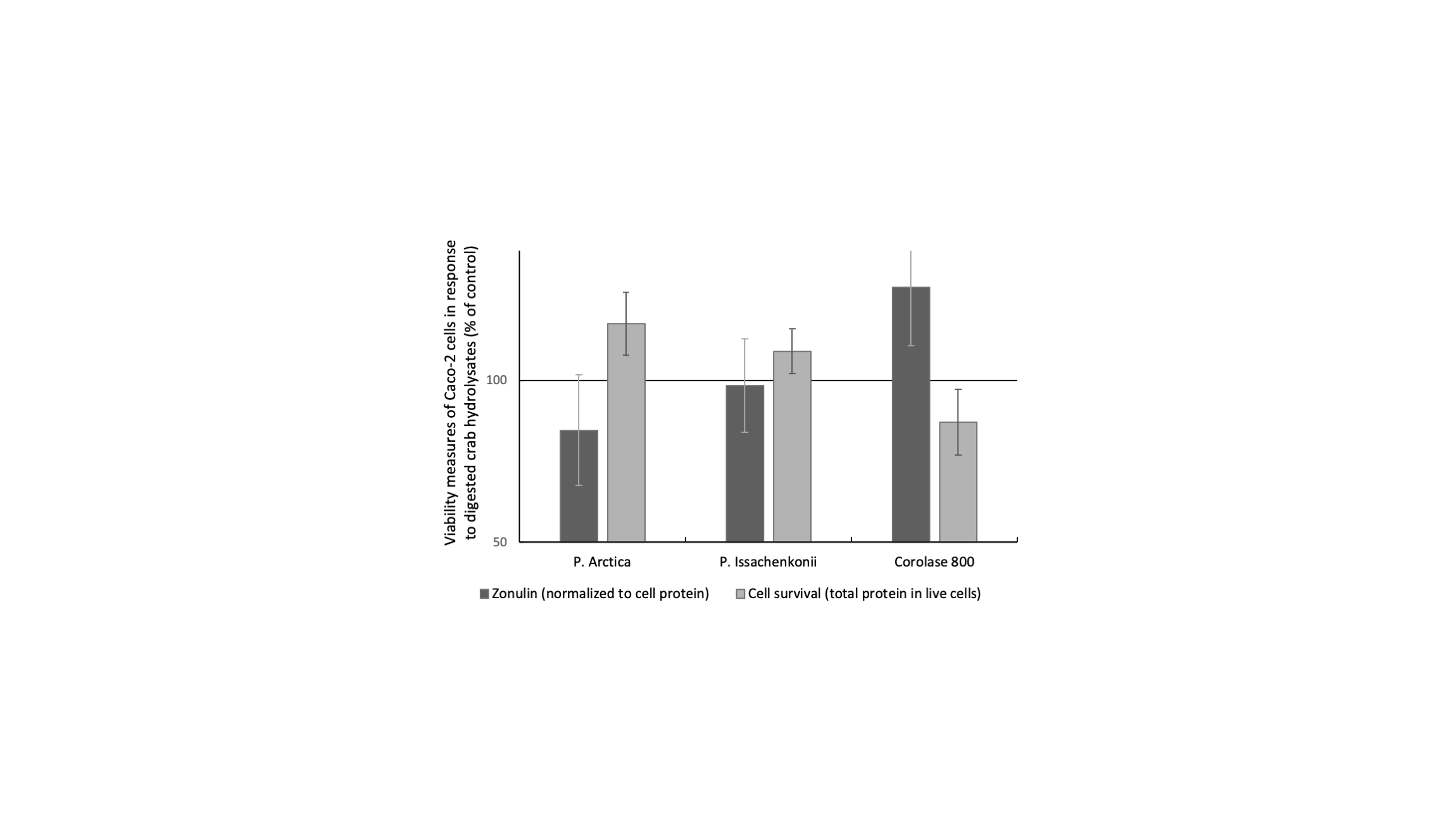

Supplement: Supplementary Figure 1 — Cell survival (estimated by total protein of live attached cells) and integrity of the Caco-cell epithelia (the tight junction marker; Zonulin) as percentage of digest blank control cells. There was no significant deviation from cell control (digestion blank, 100%) in any case. Data are means ± SD, n = 3 (Cell survival: p = 0.06, p = 0.2, p = 0.07 and Zonulin: p = 0.25, p = 0.88, p = 0.08, respectively. Changes were considered significant if p < 0.05. [file Image_1.tiff]
